# Physical activity partially mediating the social gradient in adolescent mental health

**DOI:** 10.3389/fpubh.2025.1622080

**Published:** 2025-10-13

**Authors:** Johan Dahlstrand, Qinyun Lin, Peter Friberg, Jonatan Fridolfsson, Yun Chen

**Affiliations:** ^1^School of Public Health and Community Medicine, Institute of Medicine, University of Gothenburg, Gothenburg, Sweden; ^2^Centre for Lifestyle Intervention, Institute of Medicine, University of Gothenburg, Sahlgrenska University Hospital Östra, Gothenburg, Sweden

**Keywords:** adolescent mental health, socioeconomic status, physical activity, stress, psychosomatic symptoms

## Abstract

**Objectives:**

To examine whether there is a socioeconomic status (SES) gradient in adolescent mental health problems, and if so, whether out-of-school physical activity mediates this gradient.

**Methods:**

Based on data from 1,285 adolescents in Sweden, we used linear regression analysis to examine whether the social gradient in mental health problems (stress and psychosomatic symptoms by survey) varied by SES indicators, including income, father’s and mother’s education (register data). Parameter estimates were obtained using ordinary least squares. We also investigated if out-of-school physical activity (accelerometer data) mediates these gradients by applying the potential outcomes framework for mediation analysis. This framework accounts for potential exposure-mediator interaction, and confidence intervals were calculated using bootstrapping.

**Results:**

Gradients in adolescents’ mental health problems were observed for all SES indicators, the coefficient of determination R^2^ showing the different SES indicators explained between 0.7–1.4% of stress and 0.5–1.2% of psychosomatic symptoms. Out-of-school vigorous-physical-activity (VPA) and moderate-to-vigorous-physical-activity (MVPA) partially mediated the gradients related to income in the overall sample. Specifically, VPA mediated 6.8% of the association between income and stress, and 9.2% of the association between income and psychosomatic symptoms, while MVPA mediated 5.4 and 6.6% of these associations, respectively. Regarding father’s education, VPA mediated 15.7% of the association with stress and 10.2% with psychosomatic symptoms, whereas MVPA mediated 14.6 and 8.9%, respectively. Sex-stratified analyses revealed that these mediation effects were statistically significant only among females. For mother’s education, mediation effects were observed exclusively in the female subgroup. VPA mediated 24.1% of the association with stress and 24.0% with psychosomatic symptoms, while MVPA mediated 20.8 and 21.5% of these associations, respectively.

**Conclusion:**

There are social gradients in adolescents’ mental health problems based on income and parents’ education, and these gradients appear to be partially mediated via out-of-school VPA and MVPA, predominantly among females.

## Introduction

From 1985/1986 to 2021/2022, there was a secular trend of increasing levels of self-reported psychosomatic symptoms among 13 and 15-year-old adolescents in Sweden (1). Psychosomatic symptoms among adolescents increase the risk of mental disorders such as depression and anxiety symptoms later in life (2, 3). Adolescents’ self-reported psychosomatic symptoms are also strongly correlated with stress (4).

Reports have highlighted a rise in income inequalities in Sweden since the 1980s, particularly when compared to other countries (5, 6). Sweden had relatively low level of income inequalities but is now close to the European average. This is related to increases in capital income, as labor income has become more equal (5).

Evidence shows social gradients in mental health with most studies reporting a negative relationship between socioeconomic status (SES) and youth mental health (7), indicating vicious cycles of deprivation and mental health problems through social causation and health selection.

For Nordic countries, studies on secular trends in the association between family finances and adolescent mental health have reported varying results (8, 9). In Sweden, rising worries about family finances among adolescents were shown to be associated with increasing psychosomatic problems from 1988 to 2008 (8). In Norway, rising psychological distress among adolescents from 2014 to 2018 was in tandem with stable socioeconomic inequalities (9).

Income is one of three components to consider when studying SES, alongside education and social class (10). These components are not interchangeable (10), as each indicator measures different phenomena and is associated with different pathways and causal processes. Parental income and education have been shown to be generally more important relative to parental unemployment and low occupational status (7), and income to be a strong predictor of adolescent health outcomes, while parental education is associated with child-health related behaviors (11).

Adding to the complexity, variations may depend on gender. For example, stress in girls shows a stronger social gradient with parents’ education, while income has a stronger impact on boys (12). Therefore, both parental income and education needs to be included when studying SES-related differences in adolescents’ health outcomes and behaviors.

Concurrently, insufficient physical activity (PA) among youth parallels the rising trend of mental health problems in children and adolescents. Between 2001/2002 and 2021/2022, approximately 10 to 25% of 11-, 13-, and 15-year-olds in Sweden met the World Health Organization (WHO) recommendations (1) of at least 1 h of moderate to vigorous physical activity (MVPA) per day on average (13). This is in line with studies using global data, which found 81.0% of adolescents reporting insufficient PA in 2016. Notably, physical inactivity significantly decreased among boys between 2001 to 2016, while it remained unchanged for girls (14). At this rate, WHO’s target to reduce global physical inactivity by 15% by 2030, relative to 2016 (15), will not be achieved (14).

Generally, prior evidence has shown a small negative effect of exercise interventions on psychological ill-being, including stress (16). Specifically, methodologically advanced accelerometer studies have utilized multivariate pattern analysis to investigate the relationship between adolescent PA intensity spectrum and metabolic health, providing more detailed insights into these associations (17). Those comparing different health aspects have shown that while indicators of cardiovascular risk were negatively associated with a broad range of PA intensities and positively associated with sedentary time (SED), only high intensities of PA were negatively associated with stress and psychosomatic symptoms (18).

Other studies using survey data have confirmed the mental health benefits of high intensity PA, such as vigorous physical activity (VPA) (19).

There are also evidence suggesting social gradients in adolescent PA, with higher SES linked to more PA (20). Another study found that adolescents reporting medium to high SES had less SED and engaged in more high-intensive PA measured by accelerometers than those reporting low SES (18). Specifically, low SES adolescents were shown to have lower levels of MVPA during out-of-school time, but not during school time (21).

Given the social gradient in mental health and the links between SES, PA, and mental health, could PA serve as a pathway for the social gradient in mental health? Existing evidence remains rudimentary. One study, based on a marginal structural model, found that simulated increases in MVPA in 7-year-olds would mediate small decreases in socioeconomic inequalities in mental health problems 4 years later (22). Another study, which used self-reported data on SES, PA and quality of life, investigated the mediating role of PA in the association between SES and adolescent quality of life using a counterfactual approach. The findings indicated that adherence to PA guidelines mediated SES-related differences in adolescent quality of life (23). Although this study was based on adolescents rather than children, it measured PA based on self-reporting questionnaire instead of objective accelerometer data. There appears to be a knowledge gap when it comes to accurately estimate the mediation effect of PA on the social gradient of adolescent mental health.

The secular trend in rising psychosomatic symptoms and related stress among adolescents in Sweden urge us to better understand what is needed to change the current dismal trajectory. We need to identify the resilience factors and determine how to ensure that all adolescents can take advantage of them. Prior rigorous evidence on the potential mediation role of PA on the social gradient in adolescent mental health is lacking.

To address this gap, we have placed this study within the framework of the stress process model (24). This theoretical model, supported by empirical evidence, suggests that stress may be provoked by chronic life strains, such as SES, being unfavorably accentuated by life events. According to the model, personal resources, such as PA, can influence the stress process by serving a coping mechanism in managing stress and stress-related symptoms. In our adaptation of this model, participation in out-of-school PA is considered to be partially shaped by family resources. These resources may be financial, potentially limiting access to self-funded exercise and sports activities to varying degrees, or related to parental knowledge about the health benefits of PA. Both factors may influence adolescents’ levels of PA, which in turn may affect their mental health outcomes. This study has two aims: 1) to explore whether the SES-related gradient in stress and psychosomatic symptoms among 13-year-olds varied depending on the SES indicators used; and 2) to test the hypothesis that this gradient was mediated by out-of-school PA and SED measured by accelerometer. We performed sex-specific analyses as previous studies have shown that there are sex-related differences in stress and psychosomatic symptoms (4), as well as in out-of-school VPA and MVPA (21).

## Methods

### Data and characteristics of participants

The data is drawn from the baseline survey and examinations of a cohort study in Western Sweden. Participants were seventh grade students recruited from 54 schools in 16 municipalities in Region Västra Götaland during 2015–2019. Data collection was performed at schools having agreed to participate in the study. All students in the classes who accepted invitation to participate and provided written consent by their guardian(s) and themselves were able to participate in the study. Out of a total of 5,084 students invited, 2,283 accepted to join the study. The cohort has previously been verified to be representative for the Swedish population of 13-year-olds (4). Ethics approval for the study had been granted by the Ethics Committee of the Sahlgrenska Academy, University of Gothenburg.

Data collection took place during the whole school year, beginning late August each year, and ending early June, with breaks for school holidays, including Christmas and New Year’s holiday as a longer break.

#### Exposure variables

Family SES, was measured using register data from Statistics Sweden on the income and education levels of mothers and fathers, collected for the year prior to the baseline examination.

Income was measured as the household disposable income in relation to living standard, referred to as equivalized disposable income or economic standard (5). This variable includes capital income, which is the main cause of current income disparities in Sweden. Disposable income in relation to living standard level was based on the parents’ disposable income adjusted for household size (see the Supplementary file for details).

Parental education was dichotomized separately for fathers and mothers, with a value of 1 indicating post-secondary education, and 0 representing lower levels of education achieved.

#### Mediators

The PA variables used in this study were based on accelerometer data collected using ActiGraph GT3X + (ActiGraph, Pensacola, Florida). Raw accelerometer data from the vertical axis was processed to ActiGraph counts using an epoch length of 3 s. Non-wear time was defined as at least 60 consecutive minutes of zero counts with the allowance of up to 2 min of movement below the sedentary threshold. Most adolescents provided valid data (n = 1,285), defined as wearing the device at least 10 h per day for at least 4 days.

The intensity categories used in this study included SED, light physical activity (LPA), moderate physical activity (MPA), MVPA and VPA, using Evenson’s cut points (25). See the Supplementary file for details. Minutes per day at each intensity level were standardized as 60 min/day for SED and LPA, and 15 min/day for VPA, MPA and MVPA, respectively, in the regression models. Only PA during out-of-school time was analyzed, defined as weekdays from 07:00–08:00 and 16:00–23:00, and weekends from 07:00–23:00. Supplementary Table S1 compares participants with and without valid PA data.

#### Outcome variables

Stress was estimated based on the perceived stress scale with 10 items (26), with 6 being questions on how frequently during the last month participants perceived stressful situations. The other 4 items ask questions about how frequently during the last month participants managed stress well. The answer alternatives ranged from 0 (never) to 4 (very often). The values on the stress management items were reversed before all 10 items were aggregated into a score ranging from 0 (no stress) to 40 (maximum stress). The perceived stress scale with 10 items has been validated previously for Swedish use (27).

To measure psychosomatic symptoms, we used the Psychosomatic Problem Scale with 8 items. Questions estimate the frequency of eight psychosomatic symptoms during the last 6 months, including having difficulty in falling asleep and having headache, ranging from 0 (never) to 4 (always). The total score for 8 items were calculated, ranging from 0 (no symptoms) to 32 (maximum symptoms). The validity of the scale has been confirmed by a previous study using Swedish data (28).

For both variables, if an item was missing, we replaced it with the intrapersonal mean of the other items of the respective scales. The Cronbach’s alpha coefficient was 0.812 for the perceived stress scale and 0.837 for the Psychosomatic Problem Scale, indicating that both scales have high internal consistency reliability. The item-total correlations were 0.491 ± 0.082 for the perceived stress scale and 0.566 ± 0.030 for the Psychosomatic Problem Scale. Based on these results we may conclude that our method of imputation for subjects with one missing item is acceptable (29).

#### Confounders

For the mediation analyses we used age and sex at birth as confounders, excluding sex when stratifying by sex. Age is the age at physical examination.

For our sensitivity analyses, we added immigrant background, family structure and season. Immigrant background is based on register data, and follows the criteria by Statistics Sweden, whereby one has an immigrant background if you were born outside Sweden, and or both of your parents were born outside of Sweden. Family structure is dichotomous, that you either live with or you do not live with both parents. Season refers to the date of the examination, dichotomizing participants into either winter category (1^st^ October to 31^st^ March) or summer category (1^st^ April to 30^th^ September).

### Statistical analysis

**Figure 1:**
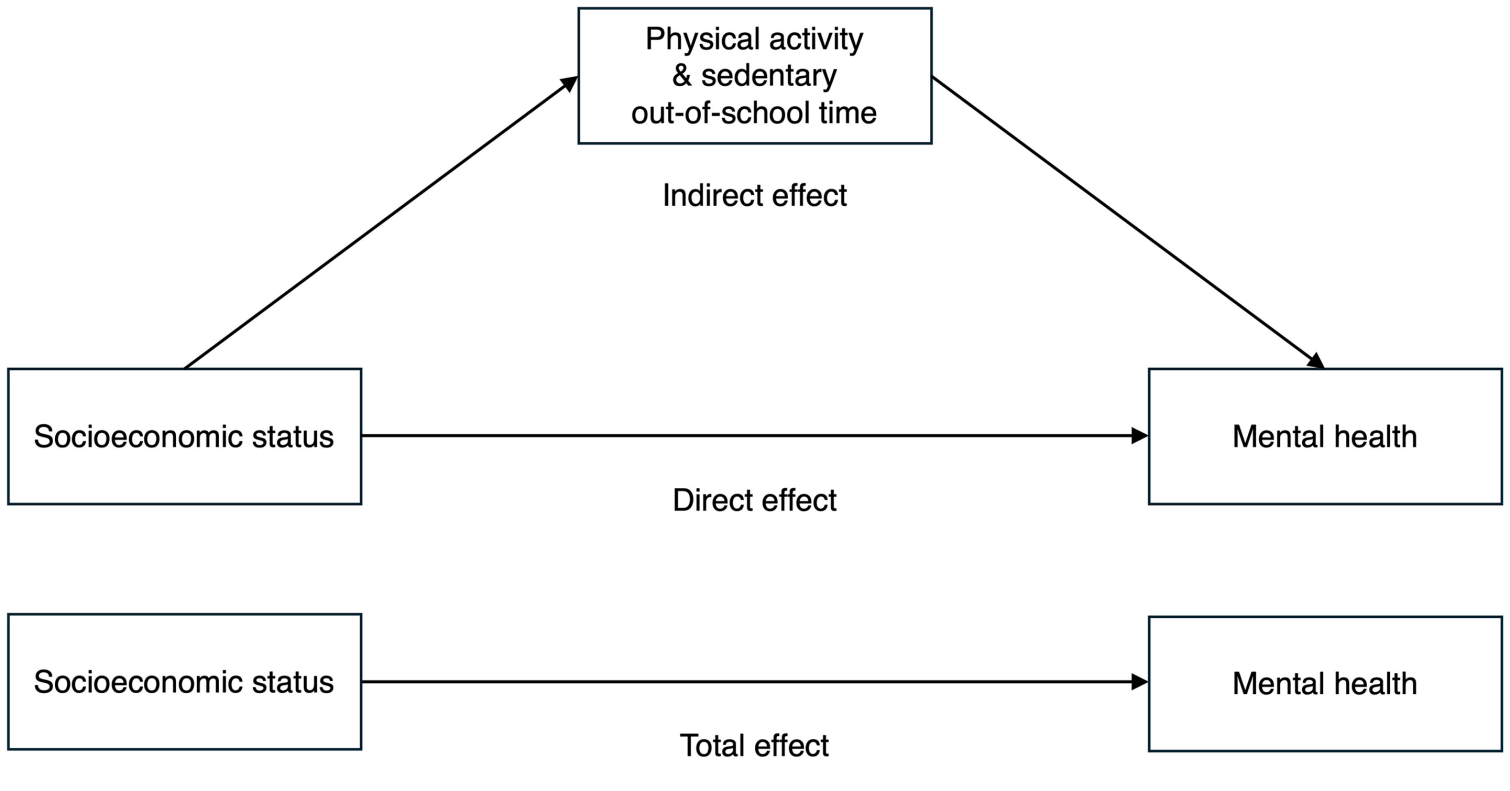
Overview of the mediation model. Study of adolescence resilience and stress, Sweden, 2015–2019.

The total effect of SES on mental health was first estimated using ordinary least squares linear regression, using each SES indicator as a separate predictor of stress and psychosomatic symptoms. We also examined the potential mediation roles of PA and SED between SES and mental health. The simple mediation model with one mediator is illustrated in Figure 1.

We used the potential outcomes framework in the mediation analysis, a modern approach that emphasizes causal estimation and necessary assumptions (30). This method also accounts for potential exposure-mediator interaction, enhancing the understanding of the conditional nature of the mediation process (31). Such insights have practical policy implications, as it may identify conditions under which a mediation process is effective. We used model 4 of the PROCESS macro (version 4.3) for IBM SPSS Statistics, which relies on regression-based estimation of the mediator and outcome models and is consistent with the counterfactual framework, allowing for exposure-mediator interaction (32). It estimates parameters using ordinary least squares regression (33) and calculates confidence intervals (CI) for mediation effects via non-parametric bootstrapping, which avoids the assumption of normality in the sampling distribution of indirect effects and is recommended practice in mediation analysis (34, 35). For bootstrapping we used 10,000 resamples. See the Supplementary file for details.

Our study includes one exposure variable measured on a continuous scale: disposable income relative to the living standard level. To estimate and interpret its effect, we selected the minimum living standard level (100) as the reference point, which is most relevant and appropriate for the Swedish context. While not a group in itself, we refer to this reference point as the low-income group (i.e., the control group). For comparison, we chose the third quartile (346.25) as the counterfactual value, representing an improved state or intervention. This value corresponds to the median of the higher-income half of the participants, which we will refer to as the high-income group.

For mediation analysis, we reported the natural indirect effect (NIE), total effect (TE), natural direct effect (NDE), and controlled direct effect (CDE(m)), each defined as the difference between two potential outcomes. NDE compares treatment vs. control group while fixing the mediator at the level of the control group. For this study, NDE represents the impact of moving from the low-income group to the high-income group on mental health outcomes while keeping PA at the level of the low-income group.

CDE(m) captures the average change in the outcome when the mediator is kept constant at a specific level m in the population, but the treatment level changes (31). Here, the treatment shift represents a change in the SES, specifically from the reference level (100) of the low-income group, to the third quartile (346.25), representing the high-income group. Meanwhile, m is set at the observed median PA intensity of the high-income group. In other words, CDE estimates the difference in the mental health outcome when SES increases from 100 to 346.25 while everyone’s PA intensity is fixed at the median value of the high-income group (i.e., 0.9393 for VPA, 1.9804 for MVPA, 0.9873 for MPA, 0.9854 for LPA and 8.1136 for SED).

We also calculated two important metrics: proportion mediated (PM) and proportion eliminated (PE) (31) to help assess the importance of the mediation process. These metrics were derived from the NIE, TE, and CDE(m) as below (31).


$$\mathrm{PM}=\frac{\mathrm{NIE}}{\mathrm{TE}}$$

$$\mathrm{PE}(m)=\frac{\mathrm{TE}-\mathrm{CDE}(m)}{\mathrm{TE}}$$

Calculating PM allows for a comparison of the NIE with TE. In relation to NIE, PM provides insights into the significance of PA in mediating the relationship between SES and mental health outcomes. However, neither PM nor NIE can directly tell the effect of intervening on PA. To address this, we use PE(m), which indicates the proportion of SES’ total effect on the mental health outcome that could be eliminated by intervening the mediator PA to a fixed level m for all subjects (31).

To assess the statistical significance of the mediation, we report the CI for NIE (31). We calculated PM and PE when TE, NIE, NDE and CDE(m) were all statistically significant and had the same sign (36). We use 5% as the significance level. All analyses were performed in IBM SPSS Statistics for Macintosh (version 29.0, Armonk, NY: IBM Corp).

## Results

### Social gradient in mental health

The mean household disposable income of the study participants was 303, more than three times the minimum living standard of 100 (Table 1). Only 2.3% of participants have a living standard below 100.

**Table 1:** Description of participants with valid accelerometer data.

Variables	*N*	%/Mean ± SD
Age (year)	1,284	13.6 ± 0.4
Male	1,284	41.3%
Immigrant background^a^	1,283	17.4%
Income^b^	1,268	303.0 ± 189.9
Father post-secondary education	1,114	51.9%
Mother post-secondary education	1,250	63.6%
VPA (min/day)	1,284	15.3 ± 9.6
MVPA (min/day)	1,284	30.8 ± 14.8
MPA (min/day)	1,284	15.5 ± 6.8
LPA (min/day)	1,284	60.0 ± 17.8
SED (min/day)	1,284	487.5 ± 56.5
Stress (scale 0–40)	1,284	15.3 ± 6.2
Psychosomatic symptoms (scale 0–32)	1,283	11.4 ± 5.4

Continuous variables reported as mean ± SD, binary variables reported as share in percent. SD, Standard deviations; VPA, Vigorous physical activity, MVPA, Moderate-to-vigorous physical activity; MPA, Moderate physical activity; LPA, Light physical activity; SED, Sedentary time.

a Immigrant background is based on register data of Statistics Sweden, who defines an immigrant to be a person born abroad and / or with both parents born abroad.

b Equivalized household disposable income ratio: mean percentage points of minimum living standard (set minimum living standard = 100).

When regressing health outcomes on income, both models showed statistically significant negative coefficients, i.e., household disposable income was negatively associated with stress and psychosomatic symptoms. The sex-specific analysis showed that for stress, the associations remained statistically significant in males but not in females, while for psychosomatic symptoms, the associations remained statistically significant in females but not in males (Table 2).

**Table 2:** Mental health problems regressed on socioeconomic status indicators.

Estimates	Stress			Psychosomatic symptoms		
	All	Males	Females	All	Males	Females
Income						
B (std. B)^a^	−0.003 (−0.100)	−0.004 (−0.147)	−0.002 (−0.064)	−0.002 (−0.078)	−0.002 (−0.074)	−0.002 (−0.076)
95% CI for B	−0.005, −0.001	−0.007, −0.002	−0.004, 0.000	−0.004, −0.001	−0.004, 0.000	−0.004, 0.000
*p*-value	<0.001	<0.001	0.08	0.005	0.091	0.039
R^2^^b^	0.009	0.02	0.003	0.005	0.004	0.004
Father’s education						
B (std. B)^a^	−1.086 (−0.089)	−1.235 (−0.106)	−0.690 (−0.057)	−1.155 (−0.108)	−1.243 (−0.123)	−0.780 (−0.074)
95% CI for B	−1.799, −0.374	−2.295, −0.174	−1.619, 0.239	−1.784,-0.527	−2.161, −0.324	−1.595, 0.034
*p*-value	0.003	0.023	0.145	<0.001	0.008	0.06
R^2^^b^	0.007	0.009	0.002	0.011	0.013	0.004
Mother’s education						
B (std. B)^a^	−1.537 (−0.120)	−1.803 (−0.148)	−1.139 (−0.090)	−1.263 (−0.111)	−1.465 (−0.137)	−0.894 (−0.080)
95% CI for B	−2.245, −0.828	−2.849, −0.757	−2.059, −0.220	−1.891, −0.635	−2.387, −0.543	−1.697, −0.090
*p*-value	<0.001	<0.001	0.015	<0.001	0.002	0.029
R^2^^b^	0.014	0.02	0.007	0.012	0.017	0.005

CI, confidence interval.

a Unstandardized beta coefficient (Standardized beta coefficient).

b Adjusted coefficient of determination.

We found that father’s education level was negatively associated with both stress and psychosomatic symptoms, with the associations primarily observed in males (Table 2).

Using mother’s education as an SES indicator, we found a negative association with stress and psychosomatic symptoms, which remained statistically significant for both males and females in sex-specific analysis (Table 2).

These SES indicators explained a small proportion of the variations in health outcomes, with adjusted R^2^ ranging 0.7–1.4% for stress and 0.5–1.2% for psychosomatic symptoms across the whole sample. Notably, the explained variations was slightly higher in males than in females. For example, mother’s education explained 2.0 and 1.7% of variations of stress and psychosomatic symptoms, respectively, in males, compared to 0.7 and 0.5% in females.

To examine the independence of the SES indicators, we ran extended models by simultaneously regressing health outcome on all three SES indicators. Considering the observed correlations among the three SES indicators (Supplementary Table S2), we first examined multicollinearity and found that the variance inflation factor ranged from 1.075 to 1.185, indicating a low risk of multicollinearity. The extended linear regression model for stress showed a significant coefficient only for mother’s education, but not for the other two SES indicators. In the extended model for psychosomatic symptoms, a statistically significant coefficient was observed exclusively for father’s education. The coefficients of determination (R^2^) increased slightly, accounting for an estimated 1.5% of the variance for both health outcomes.

### PA/SED as a mediator for the association between SES and health outcomes

#### Income

Negative associations between income and mental health problems were found in the TE estimates (Supplementary Tables S3, S4), confirming the social gradient in mental health.

**Figure 2:**
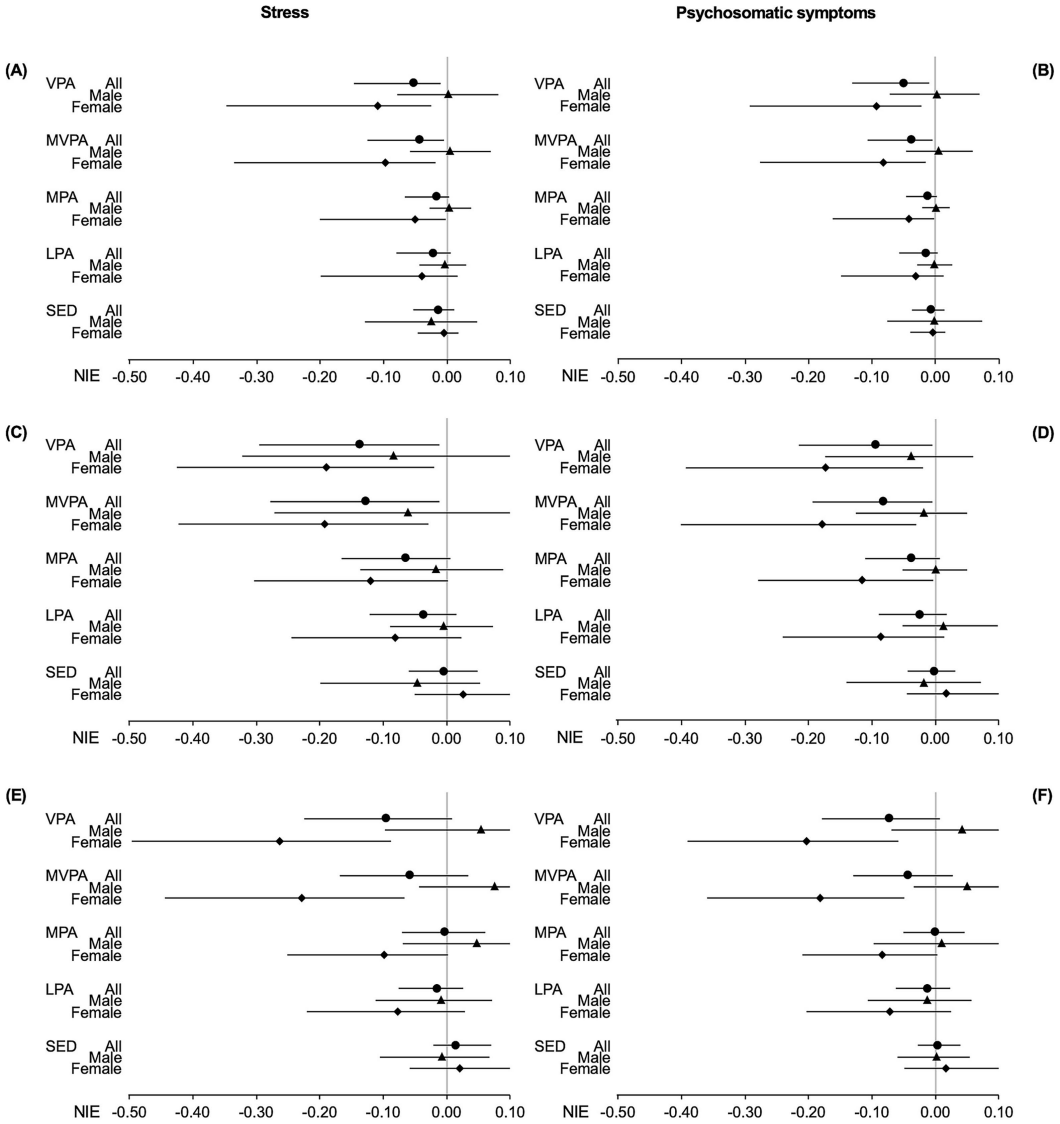
Forest plot of the natural indirect effect of the mediation analysis for the relation of parents’ income **(A,B)**, father’s education **(C,D)** and mother’s education **(E,F)**, with stress **(A,C,E)** and psychosomatic symptoms **(B,D,F)** mediated via out-of-school physical activity intensities and sedentary time. Study of adolescence resilience and stress, Sweden, 2015–2019. Dots (all), triangles (males) and diamonds (females) with bars are standardized beta coefficient with 95% confidence interval; VPA, vigorous physical activity; MVPA, moderate to vigorous physical activity; MPA, moderate physical activity; LPA, light physical activity; SED, sedentary time; NIE, natural indirect effect.

Looking at NIE, we observed that both VPA and MVPA significantly mediated the associations of income with both health outcomes (Figures 2A,B; Supplementary Tables S3, S4). Adolescents with higher parental income were more likely to have higher level of VPA or MVPA, which in turn was associated with lower stress and psychosomatic symptoms.

We found statistically significant negative estimates for CDE. This means that even if we intervene for all adolescents to have the median amount of VPA or MVPA of the high-income group, lower income is still significantly associated with higher stress or psychosomatic symptoms.

Given significant TE and NIE for VPA and MVPA, we calculated the PM and PE. For stress, a PM of 0.068 with VPA as mediator was found, suggesting that VPA may mediate 6.8% of the association between income and stress. The accompanying PE of 0.097 suggests that 9.7% of the income’s impact on stress could be eliminated if all adolescents would adhere to the high-income group’s median VPA level of 14.1 min per day. The PM and PE with MVPA as mediator were 5.4 and 6.6%, respectively. For psychosomatic symptoms, PM and PE with VPA as mediator were 9.2 and 15.1% respectively, while PM and PE with MVPA as mediator were 6.6 and 9.1%, respectively.

To investigate sex differences, we performed mediation analysis for males and females separately. For males, none of the PA intensities or SED mediated the association between either income and stress or income and psychosomatic symptoms. For females, the models with VPA, MVPA and MPA as mediators all had significant NIE estimates, suggesting significant mediation processes. However, only the model with MPA as mediator for the association between income and psychosomatic symptoms had statistically significant TE, associated with a PM at 7.1%. As this model has a CDE greater than TE, it results in a negative PE, which is not meaningful to interpret (36).

#### Father’s education

For the associations between fathers’ education and health outcomes, we found statistically significant mediation of VPA and MVPA for stress (Figure 2C; Supplementary Table S5) and psychosomatic symptoms (Figure 2D; Supplementary Table S6). The calculated PM suggested that VPA and MVPA may mediate 15.7 and 14.6% of the association between fathers’ education and stress, respectively. Meanwhile, VPA and MVPA mediated 10.2 and 8.9% of the association between father’s education and psychosomatic symptoms, respectively.

We found statistically significant CDE for mediation models (Supplementary Tables S5, S6). For stress, the calculated PE was 0.170 for VPA and 0.149 for MVPA, indicating that 17.0 and 14.9% of the impact of stress could be eliminated by keeping VPA and MVPA, respectively, at the median level of VPA and MVPA of the adolescents with fathers having post-secondary education (median levels of VPA and MVPA are 14.6 and 29.9 min per day, respectively). For psychosomatic symptoms, the calculated PE was 0.106 for VPA and 0.091 for MVPA.

Sex-specific analysis showed that for males, none of the PA intensities or SED had statistically significant NIE (Figures 2C,D; Supplementary Tables S5, S6), meaning neither PA nor SED significantly mediates the associations between fathers’ education and health outcomes. In contrast, for females, models for stress revealed statistically significant NIE for VPA and MVPA. Additionally, models for psychosomatic symptoms for females showed statistically significant NIE for VPA, MVPA and MPA. As none of these models had any statistically significant TE or CDE(m), we did not calculate PM or PE.

#### Mother’s education

For the association between mothers’ education and health outcomes, we did not find any statistically significant estimates of NIE, suggesting no significant mediation process through PA or SED, when analyzing the whole sample or only males (Figures 2E,F; Supplementary Tables S7, S8). When analyzing only females, we found that both VPA and MVPA significantly mediated the associations between mothers’ education and stress (Figure 2E; Supplementary Table S7); and between mothers’ education and psychosomatic symptoms (Figure 2F; Supplementary Table S8). However, as NDE was not statistically significant for VPA and MVPA, and CDE was not significant for MPA, LPA and SED, for the stress models, it makes no sense to calculate PM or PE. We found similar results for psychosomatic symptoms, making calculations of PM and PE irrelevant.

#### Sensitivity analysis

For the mediation analysis, we used age and sex as confounders (age for sex separated analyses). Whilst immigrant background and family structure may plausibly confound associations between SES, PA and mental health problems, we conceptualized them as part of a broader definition of SES and follow standard practice in operationalizing SES via education and income, which are well-measured and widely used proxies. To avoid overadjustment, these variables were therefore not included in the main models.

At the same time, we recognize that immigrant background and family structure may have SES-independent effects on PA and mental health problems. We also found that there were more sedentary time and less LPA, MPA, MVPA and VPA for the winter category, relative to summer during out-of-school time. Independent samples t-tests showed that while the difference in sedentary time was not statistically significant, the differences in LPA, MPA, MVPA and VPA were. As a seasonal effect is plausible also for mental health problems, we conducted sensitivity analyses that included all three variables immigrant background, family structure and season of assessment.

We re-ran all models including immigrant background, family structure, and season of assessment as additional covariates. The overall pattern of results remained consistent with the original analyses, particularly for females. Some additional effects reached statistical significance when season was included, which likely reflects improved precision due to season accounting for variance in both the mediator and outcome. Importantly, no results reversed direction, and the key conclusions of the study remain unchanged.

This shows that the results were largely unchanged — reinforcing the robustness of our findings.

## Discussion

The present study shows a social gradient in adolescent’s mental health problems, irrespective whether household disposable income, father’s education or mother’s education is used as a measure of SES. The social gradient varies by sex, with males showing a stronger gradient related to parents’ education.

Further, out-of-school VPA and MVPA appear to partially mediate these social gradients in mental health problems, especially among females. Our study provides new evidence regarding the role of PA in the social gradient in adolescent mental health problems and highlight the importance in considering the sex-related difference when formulating policy and planning interventions.

Our findings on social gradients in mental health problems among adolescents are in line with reviews (7) and studies on recent trends in Sweden (37). Our study shows that the income-related gradient does not remain statistically significant in the extended model that included all three SES indicators simultaneously. As expected, the income-related gradient seems to be influenced by parent’s education, corroborated by statistically significant correlation between income and education (Supplementary Table S2). Furthermore, we found a social gradient in stress among females, but only when using mother’s education as the SES indicator. This aligns with previous study showing that for females, parents’ education as a SES indicator shows a stronger social gradient in stress than income (12). However, we did not find any evidence that income would show a stronger social gradient in stress among males than parents’ education, as that study did. The diverging results depending on SES indicator used align with evidence showing indicators are not interchangeable (10).

Interestingly, we demonstrated that the social gradient in adolescents’ mental health problems appear to be partially mediated by out-of-school PA, especially with income and father’s education as SES indicators. Mediation is only found for VPA and MVPA, but not for lower intensities or SED. Our findings appear to be in line with evidence (22) reporting slight decreases in internalizing problems when increasing MVPA to the WHO recommendations of 60 min/day. However, the same study showed a slight reverse social gradient in PA based on income, which is opposite to what our data show when it comes to the correlation between income and VPA/MVPA. The difference may be explained by the exclusion of school-time PA in the present study. The schools could provide similar opportunities for PA to students with varying SES, potentially masking SES-related disparities. Additionally, the simulation study (22) was carried out on children rather than adolescents, with internalizing problems reported by their parents. Meanwhile, our results align with those of another study that investigated PA as a mediator for the social gradient in quality of life, where significant mediation via PA was found (23).

The mediation of the social gradients in stress and psychosomatic symptoms through PA, using income- and father’s education as SES indicators, appear to be driven by females. When the data is separated by sex, mediation is not statistically significant for males. The influence of females is especially evident in mediation models using the mother’s education as an SES indicator.

One explanation for these sex-specific observations could be sociocultural factors, e.g., marked differences in out-of-school exercise and sports between females and males. Evidence suggests that public subsidies at national and local levels have traditionally been biased toward team sports arranged by civil society, where boys tend to participate more frequently. In contrast, girls are more likely to participate in individual sports, which are often commercially organized and lack public funding (38). This could mean that while boys have equal access to play ice-hockey in a rink financed by the local council, girls are horseback riding only to the extent that their parents can afford buying and maintaining a horse. This highlights how chronic life strains, as described in the stress process model (24), can influence adolescents thorough the *material* realities of socioeconomic status, which can limit access to preferred forms of physical activity due to financial constraints. Another pathway might be through the *status* component of socioeconomic status, affecting self-confidence and self-perception (39, 40).

### Strengths and limitations

This study is strengthened by a relatively large cohort of adolescents within a narrow age range, with SES measured using register data, offering greater accuracy than self-reported affluence. The use of accelerometers to measure PA and SED also enhances accuracy compared to self-reports. Additionally, the mediation analyses use a modern approach, accounting for exposure-mediator interactions. Furthermore, this study consistently included sex separate analyses, and has undertaken sensitivity analyses for a several other variables. Considering the results of our sex separate analyses, we recommend researchers to include that in future studies. Also, the sensitivity analyses including additional confounders strengthen the robustness of the findings. Based on our results, we recommend that researchers studying physical activity in geographies similar to Western Sweden take potential seasonal effects into account.

Sweden is a high-income country and a welfare-based society, making it unclear how much this evidence lends itself to be generalized to other populations around the world, mostly living in other contexts. Specifically, this could relate to the distinct bias of Swedish public funding of out-of-school exercise and sports discussed above, potentially creating sex-specific inequalities. Thus, we recommend future research to extend similar analyses to cohorts of adolescents in other contexts, not least middle-income countries where most adolescents live.

Self-report surveys of mental health indicators are inherently subjective and may introduce biases leading to inaccurate portrayal of mental health problems, and thereby limit the validity of the study results. These biases could be both premeditated, e.g., social desirability bias, or caused by lack of capacity, e.g., recall bias. However, the specific scales used have previously been validated, as described in the Methods section. Further, the cohort was preceded by a pilot study including 177 students in 7^th^ grade, which tested the scales. To minimize recall bias, participants were instructed to report symptoms and experiences within clearly defined and recent timeframes. Additionally, participants were assured of complete anonymity and confidentiality in their responses, which is known to reduce social desirability bias, as individuals are more likely to report sensitive information honestly when they feel secure. Finally, we used confounders, age and sex, in the main analyses, as well as conducted sensitivity analyses by controlling for potential additional confounders (immigrant background, family structure and season) to assess the robustness of our findings.

Mediation models are used for causal explanations, which means mediator M (physical activity) needs to be found chronologically between X (SES) and Y (mental health) (33). The time lapse should also be long enough for the effect to occur, and short enough to be still observable. The register data for the exposure variable has been collected to accommodate these principles. Additionally, although SES and mental health can influence each other (41), this is not applicable here as SES is about parental data while the health outcome pertains to adolescents.

The collection of questionnaire data and use of accelerometers took place during the same period; therefore, the chronological order of mediator (PA and SED) and outcomes (stress and psychosomatic symptoms) cannot be guaranteed. This is a weakness of our study, as there is evidence that mental health may indeed have an effect on PA (19). However, as we have used data from an observational study, the PA patterns and SED measured via accelerometers reflect participants’ typical behaviors during that time in life, rather than behaviors influenced by an intervention. While we view this a minor limitation, we encourage future studies to investigate this further using longitudinal data that distinguish the temporal sequence between physical activity and health outcomes, allowing for stronger inferences about causality.

The statistical analyses have not considered clustering effects, which is a limitation as students were nested within schools. However, in a related study, we previously examined this in a related study and found that only a small proportion of the variance in stress and psychosomatic symptoms—approximately 2.5%—was attributable to differences between schools. This suggests that most variation occurs at the individual level. Given this minimal between-school variance, the inclusion of random effects for school clustering would likely have a negligible impact on model performance or inference. This is consistent with established guidelines indicating that when intraclass correlation coefficients are low (typically below 0.05), the benefits of modeling clustering are limited.

## Conclusion

Disposable income and father’s and mother’s education all constitute social gradients in stress and psychosomatic symptoms among adolescents. Further, only higher intensities of PA appear to play a mediation role in the social gradient in stress and psychosomatic symptoms, and this mediation was mainly found in female adolescents.

## Data Availability

The datasets presented in this article are not publicly available because the STARS study comprises sensitive personal data and the register data from Statistics Sweden, which have been linked to the STARS study, contain personal data. The data are therefore protected by the Swedish Public Access and Secrecy Act and access to these datasets are restricted. However, researchers who are interested in collaboration on the STARS data should contact the principal investigator Professor Peter Friberg (peter.friberg@mednet.gu.se) or Yun Chen (yun.chen@wlab.gu.se).
