# Supplementary material for: Physical activity partially mediating the social gradient in adolescent mental health
Source: Front Public Health. 2025 Oct 13;13:1622080. doi: 10.3389/fpubh.2025.1622080 (PMC12554433; doi:10.3389/fpubh.2025.1622080)
Supplement: Supplementary file 1 [file Data_Sheet_1.pdf]

Physical activity partially mediating the social gradient in adolescent mental health

Supplementary file

Methods

Mediators

Evenson's cut-points for sedentary time (SED), light physical activity (LPA), moderate physical activity (MPA), moderate and vigorous physical activity (MVPA) and vigorous physical activity (VPA) are widely used and have been suggested to be the most appropriate option for studies on children using ActiGraph counts (1). The Evenson cut-points, originally developed for 15-second epochs using vertical axis accelerometer counts, were defined as follows: SED (0-25 counts/15 seconds), LPA (26-573 counts/15 seconds), MPA (574-1002 counts/15 seconds), and VPA (≥1003 counts/15 seconds). As our study collected data in 3-second epochs, we adjusted these thresholds proportionally by dividing each cut-point by 5, resulting in: SED (0-5 counts/3 seconds), LPA (6-114 counts/3 seconds), MPA (115-200 counts/3 seconds), and VPA (≥201 counts/3 seconds). MVPA was calculated as the sum of time spent in MPA and VPA.

Exposure variables

The definition of "Disposable income in relation to living standard" is explained in Statistics Sweden's "Documentation of STATIV 1997-2016" (2): "Disposable income in relation to reasonable living standard, e.g., 134 percent indicates that the family has a disposable income 34 percent higher than the reasonable standard of living that is relevant given the family's size and nature.

For 1997-1999 the information on disposable income/standard of living is based on previously obtained basic information from the National Board of Health and Welfare. Disposable income is what remains for consumption and savings after taxes have been subtracted and after-tax free transfers

(subsidies) have been added to the taxable gross income. The taxable gross income constitutes salary income, income from business activity, income from capital, pension and annuity, income from hobby activity, income from privately held company as well as taxable transfers such as e.g. compensation for unemployment, sickness pay, parental subsidy, etc.

Tax designates income tax on income from work, tax on capital earnings, general person expenses, property tax, taxes on savings and person expenses for business. The concept of tax is included in the calculation of disposable income even if the negative transfers for cost-of-living subsidies and study loans have been paid back.

The tax-free transfers constitute housing subsidy, housing supplements for pensioners, child subsidy, social subsidy, subsidy advance, handicap compensation, study loan and study subsidy, compensation for those in military service as well as subsidies for small groups of recipients. Disposable income is a family concept. Several subsidies such as e.g., child subsidy, housing subsidy, and social subsidy are not given to persons but rather to the family. In the income statistics, the family is the normal observation unit. However, the STATIV database is based on the person, and it is difficult to create simple tables where the family constitutes the observation units. Almost all the other variables in the database are linked to the person, e.g. level of education and residence in Sweden.

The composition of the family changes over time. Persons separate or receive married, die, have children, etc. In longitudinal studies, the family perspective is affected by major methodological problems. We therefore want to create a concept that takes into consideration the different compositions of families yet can function at the person level also.

We have used the family's disposable income as an economic measure, but we have then used the persons in the family as observation units. As a result, even the children in a family are given a measure of their economic standing. The family's disposable income is related to the social subsidy level that, according to the model used, applies to just that family (based on the family's composition and place of residence). If the family has a disposable income that is 130 percent of the social subsidy level, that is 30 percent better than the acceptable living standard, then each member of the household is assigned that value, even the children.

In 1998 approximately one in five persons had a disposable income that was just under or just over the social subsidy level (<125 percent). However, far from everyone in this group could be regarded as poor. These persons may be e.g. students, performing military service, have private means, or during all or part of the year lived outside of Sweden. Sixty percent of those who in Statistics Sweden's standard-of-living study from 1996 had low incomes indicated that they had (<125 percent) also reported that they did not have any social subsidies and did not have any difficulty in managing their ongoing expenses either."

For adolescents with parents that have separated and where both have data on income (n=483), we calculated average of the registry data for the parents, as the data represent the household. In the case that one of the parents has missing data (n=124), the variable takes on the value of the other parent. For 45 adolescents, both parents' data were missing and thus excluded from the analysis.

Statistical analysis

For the mediation analyses, we used model 4 of the PROCESS macro (version 4.3) for IBM SPSS Statistics. The PROCESS macro estimates parameters using ordinary least squares regression (3). The macro calculates confidence intervals (CI) using bootstrapping, with this study setting resamples to 10,000 for the analyses.

The potential outcomes framework (4-6) distinguishes an individual's observed and counterfactual outcomes. For instance, when examining a treatment effect, the potential outcomes framework defines the treatment effect as the difference between two potential outcomes, namely the outcome for the individual if they are in the treatment group and the outcome for the individual if they are in the control group. Practically, only one of these outcomes is observable while the other one is counterfactual. Similarly, the modern approach defines different estimands in mediation by comparing different potential outcomes.

For example, the natural indirect effect (NIE) is defined as the difference between two potential outcomes Y_{aMa} and Y_{aMa^*}. Y_{aMa} refers to the potential outcome when exposure X is set to some level a and the mediator M takes on the value when X is equal to a. In comparison, Y_{aMa^*} refers to the potential outcome when exposure X is still set to the level a but the mediator M is set to whatever value it may take when X is equal to a^*. In other words, the NIE evaluates the effect of the treatment

X when individuals' exposure is set to some level a while the level of mediator M is changed due to X changing from level a to a^*. For dichotomous exposure variables, a and a^* typically refers to 1 and 0, respectively (e.g. treatment and control). For a continuous exposure variable, those levels need to be set prior to estimation, based on what would be relevant for the specific context.

Our modeling proceeded in two steps. First, we estimated the total effect of SES on mental health using a linear regression model: $Y_i = \gamma_0 + \gamma_1 SES_i + \gamma_2 C_i + \epsilon_{Y_i}$, where Y_i is the mental health outcome and C_i represents covariates.

Second, we implemented mediation analysis, estimated the following regression models:

Mediator model: $M_i = \alpha_0 + \alpha_1 SES_i + \alpha_2 C_i + \epsilon_{M_i}$

Outcome model: $Y_i = \beta_0 + \beta_1 SES_i + \beta_2 M_i + \beta_3 (SES_i \times M_i) + \beta_4 C_i + \epsilon_{Y_i}$

Where M_i denotes physical activity. This specification follows the counterfactual-based mediation framework and allows for exposure-mediator interaction (7).

Identification of mediation effects relies on the assumption of sequential ignorability (7). Specifically, this requires that: (1) there are no unmeasured confounders of the exposure-outcome relationship, conditional on observed covariates; (2) no unmeasured confounders of the mediator-outcome relationship, conditional on exposure and covariates; (3) no unmeasured confounders of the exposure-mediator relationship conditional on observed covariates; and (4) no mediator-outcome confounders that are themselves affected by exposure. All four assumptions are required for the identification of natural direct and indirect effects; while only the first two are needed for conditional direct effects.

References

1. Migueles JH, Cadenas-Sanchez C, Ekelund U, Nyström CD, Mora-Gonzalez J, Löf M, et al. Accelerometer Data Collection and Processing Criteria to Assess Physical Activity and Other Outcomes: A Systematic Review and Practical Considerations. Sports Med. 2017;47(9):1821-45.
2. Documentation of STATIV: 1997-2016. Statistics Sweden; 2018 23 May 2018.
3. Hayes AF. Introduction to mediation, moderation, and conditional process analysis : a regression-based approach. New York ;: The Guilford Press; 2018.
4. Rubin DB. Estimating causal effects of treatments in randomized and nonrandomized studies. Journal of educational psychology. 1974;66(5):688-701.

5. Holland PW. Statistics and Causal Inference. Journal of the American Statistical Association. 1986;81(396):945-60.
6. Holland PW. Causal Inference and Path Analysis. Sociological methodology. 1988;18:449.
7. Valeri L, VanderWeele TJ. Mediation Analysis Allowing for Exposure-Mediator Interactions and Causal Interpretation: Theoretical Assumptions and Implementation With SAS and SPSS Macros. Psychological methods. 2013;18(2):137-50.

Table S1 Comparative statistics of adolescents with or without valid accelerometer data

Variable	Adolescents with valid accelerometer data		Adolescents without valid accelerometer data		Comparing sample[a]
	N	Mean±SD or %	N	Mean±SD or %	p
Age (years)	1284	13.6±0.4	996	13.6±0.4	0.286
Sex	1284	41.3% male	996	48.5% male	<0.001
Immigrant background	1283	17.4%	991	25.7%	<0.001
Income	1268	303.0±189.9	970	307.9±537.6	0.763
Father post-2nd education	1114	51.9%	816	46.4%	0.018
Mother post-2nd education	1250	63.6%	953	56.6%	<0.001
Stress (0-40)	1284	15.3±6.2	992	15.9±6.1	0.018
Psychosomatic symptoms (0-32)	1283	11.4±5.4	994	12.0±5.9	0.010

Note: [a]Pearson Chi-square tests of Asymptotic significance (2-sided) performed for dichotomous variables, and t-tests for equality of means (2-sided) with equal variances assumed for continuous variables.

Tabel S2 Bivariate correlations between variables

Spearman's rho	1	2	3	4	5	6	7	8	9	10	11	12	13
**1. Stress**	1.000												
**2. Psychosomatic symptoms**	0.684**	1.000											
**3. VPA leisure**	-0.124**	-0.134**	1.000										
**4. MVPA leisure**	-0.111**	-0.103**	0.930**	1.000									
**5. MPA leisure**	-0.066*	-0.044	0.670**	0.887**	1.000								
**6. LPA leisure**	-0.085**	-0.069*	0.488**	0.630**	0.707**	1.000							
**7. SED leisure**	0.044	0.026	-0.232**	-0.235**	-0.186**	-0.016	1.000						
**8. Disposable income**	-0.150**	-0.141**	0.127**	0.106**	0.071*	0.069*	-0.077**	1.000					
**9. Father post-2nd education**	-0.088**	-0.112**	0.089**	0.081**	0.056	0.050	-0.011	0.356**	1.000				
**10. Mother post-2nd education**	-0.116**	-0.100**	0.068*	0.047	0.008	0.022	0.023	0.293**	0.356**	1.000			
**11. Sex**	-0.248**	-0.289**	0.102**	0.080**	0.027	0.021	-0.051*	0.007	0.040	0.025	1.000		
**12. Age**	0.062*	0.087**	0.013	0.006	-0.011	-0.062*	-0.008	0.022	-0.068*	0.002	-0.032	1.000	
**13. Immigrant background**	0.061**	-0.009	-0.100**	-0.100**	-0.084**	-0.068**	-0.012	-0.395**	-0.104**	-0.208**	0.03	0.028	1.000

*p < 0.050, **p < 0.010. Note: VPA, vigorous physical activity; MVPA, moderate-to-vigorous physical activity; MPA, moderate physical activity; LPA, light physical activity; SED, sedentary time; Father post-2nd education: 0=father has no post-secondary education, 1=father has post-secondary education; Mother post-2nd education: 0=mother has no post-secondary education, 1=mother has post-secondary education; Sex: 0=female, 1=male; Immigrant background: 0=Swedish background, 1=immigrant background

Table S3 Parents' income-related social gradient in stress and possible mediation via out-of-school time physical activity intensities and sedentary time

Mediators	All	Male	Female
**TE**			
VPA	-0.777* (-1.613,-0.296)	-0.998* (-2.263,-0.502)	-0.512 (-1.647,0.386)
MVPA	-0.792* (-1.605,-0.301)	-1.012* (-2.284,-0.498)	-0.539 (-1.637,0.372)
MPA	-0.808* (-1.611,-0.325)	-1.070* (-2.287,-0.511)	-0.566 (-1.698,0.290)
LPA	-0.851* (-1.629,-0.403)	-1.083* (-2.277,-0.535)	-0.711 (-1.909,0.045)
SED	-0.758* (-1.642,-0.262)	-1.072* (-2.227,-0.461)	-0.530 (-1.972,0.153)
**NIE**			
VPA	-0.053* (-0.147,-0.010)	0.002 (-0.079,0.080)	-0.109* (-0.348,-0.025)
MVPA	-0.043* (-0.126,-0.005)	0.005 (-0.059,0.069)	-0.098* (-0.335,-0.018)
MPA	-0.017 (-0.066,0.003)	0.003 (-0.028,0.038)	-0.051* (-0.201,-0.002)
LPA	-0.022 (-0.080,0.006)	-0.003 (-0.044,0.030)	-0.040 (-0.199,0.017)
SED	-0.014 (-0.053,0.011)	-0.025 (-0.130,0.047)	-0.005 (-0.046,0.018)
**NDE**			
VPA	-0.725* (-1.149,-0.300)	-1.000* (-1.632,-0.369)	-0.403 (-0.981,0.175)
MVPA	-0.749* (-1.175,-0.323)	-1.016 *(-1.655,-0.378)	-0.441 (-1.019,0.137)
MPA	-0.791* (-1.220,-0.363)	-1.074* (-1.717,-0.431)	-0.515 (-1.094,0.063)
LPA	-0.829* (-1.268,-0.391)	-1.081* (-1.708,-0.453)	-0.671* (-1.288,-0.054)
SED	-0.744* (-1.191,-0.297)	-1.047* (-1.723,-0.372)	-0.525 (-1.121,0.072)
**CDE**			
VPA	-0.702* (-1.126,-0.278)	-1.141* (-1.764,-0.518)	-0.433 (-1.011,0.145)
MVPA	-0.740* (-1.165,-0.314)	-1.091* (-1.713,-0.469)	-0.515 (-1.096,0.065)
MPA	-0.756* (-1.181,-0.330)	-1.093* (-1.720,-0.466)	-0.566 (-1.148,0.017)
LPA	-0.823* (-1.259,-0.386)	-1.087* (-1.716,-0.459)	-0.692* (-1.318,-0.066)
SED	-0.744* (-1.181,-0.308)	-1.058* (-1.693,-0.423)	-0.529 (-1.139,0.082)
**PM**			
VPA	0.068	-	-
MVPA	0.054	-	-
MPA	-	-	-
LPA	-	-	-
SED	-	-	-
**PE**			
VPA	0.097	-	-
MVPA	0.066	-	-
MPA	-	-	-
LPA	-	-	-
SED	-	-	-

*p < 0.050. Notes: numbers are estimates for association coefficients (95% confidence intervals). Models for all were adjusted for sex and age. Sex-specific models were adjusted for age. TE, Total Effect; NIE, Natural Indirect Effect; NDE, Natural Direct Effect; CDE, Controlled Direct Effect; PM, Proportion Mediated; PE, Proportion Eliminated; VPA, vigorous physical activity (per 15 min); MVPA, moderate-to-vigorous physical activity (per 15 min); MPA, moderate physical activity (per 15 min); LPA, light physical activity (per 60 min); SED, sedentary time (per 60 min)

Table S4 Parents' income-related social gradient in psychosomatic symptoms and possible mediation via out-of-school time physical activity intensities and sedentary time

Mediators	All	Male	Female
**TE**			
VPA	-0.537* (-1.248,-0.200)	-0.391 (-1.522,0.043)	-0.524 (-1.397,0.039)
MVPA	-0.558* (-1.269,-0.221)	-0.415 (-1.557,0.013)	-0.561 (-1.394,0.026)
MPA	-0.580* (-1.235,-0.245)	-0.489* (-1,586,-0.049)	-0.594* (-1.580,-0.094)
LPA	-0.601* (-1.309,-0.264)	-0.486* (-1.586,-0.052)	-0.736* (-1.834,-0.227)
SED	-0.490* (-1.301,-0.104)	-0.461 (-1.587,0.128)	-0.508* (-1.839,-0.007)
**NIE**			
VPA	-0.049* (-0.131,-0.010)	0.003 (-0.072,0.069)	-0.094* (-0.293,-0.022)
MVPA	-0.037* (-0.107,-0.004)	0.005 (-0.046,0.059)	-0.082* (-0.276,-0.015)
MPA	-0.012 (-0.046,0.002)	0.001 (-0.020,0.022)	-0.042* (-0.162,-0.002)
LPA	-0.015 (-0.057,0.004)	-0.001 (-0.029,0.027)	-0.031 (-0.149,0.013)
SED	-0.007 (-0.037,0.015)	-0.001 (-0.076,0.074)	-0.005 (-0.039,0.016)
**NDE**			
VPA	-0.487* (-0.859,-0.116)	-0.394 (-0.951,0.164)	-0.431 (-0.981,0.175)
MVPA	-0.521* (-0.894,-0.148)	-0.419 (-0.985,0.147)	-0.479 (-0.980,0.021)
MPA	-0.568* (-0.944,-0.193)	-0.490 (-1.060,0.081)	-0.552* (-1.054,-0.050)
LPA	-0.586* (-0.971,-0.202)	-0.486 (-1.042,0.071)	-0.705 (-1.243,-0.168)
SED	-0.483* (-0.874,-0.091)	-0.460 (-1.059,0.139)	-0.502 (-1.022,0.017)
**CDE**			
VPA	-0.456* (-0.827,-0.084)	-0.538 (-1.088,0.013)	-0.472 (-0.973,0.029)
MVPA	-0.507* (-0.880,-0.135)	-0.486 (-1.037,0.065)	-0.579* (-1.082,-0.076)
MPA	-0.518* (-0.891,-0.146)	-0.481 (-1.036,0.075)	-0.616* (-1.122,-0.111)
LPA	-0.579* (-0.962,-0.196)	-0.476 (-1.033,0.081)	-0.728* (-1.273,-0.182)
SED	-0.490* (-0.872,-0.108)	-0.475 (-1.037,0.088)	-0.493 (-1.024,0.038)
**PM**			
VPA	0.092	-	-
MVPA	0.066	-	-
MPA	-	-	0.071
LPA	-	-	-
SED	-	-	-
**PE**			
VPA	0.151	-	-
MVPA	0.091	-	-
MPA	-	-	-
LPA	-	-	-
SED	-	-	-

*p < 0.050. Notes: numbers are estimates for association coefficients (95% confidence intervals). Models for all were adjusted for sex and age. Sex-specific models were adjusted for age. TE, Total Effect; NIE, Natural Indirect Effect; NDE, Natural Direct Effect; CDE, Controlled Direct Effect; PM, Proportion Mediated; PE, Proportion Eliminated; VPA, vigorous physical activity (per 15 min); MVPA, moderate-to-vigorous physical activity (per 15 min); MPA, moderate physical activity (per 15 min); LPA, light physical activity (per 60 min); SED, sedentary time (per 60 min)

Table S5 Fathers' education-related social gradient in stress and possible mediation via out-of-school physical activity intensities and sedentary time

Mediators	All	Male	Female
**TE**			
VPA	-0.870* (-1.567,-0.158)	-1.223* (-2.296,-0.169)	-0.620 (-1.588,0.291)
MVPA	-0.870* (-1.562, -0.180)	-1.223* (-2.279,-0.176)	-0.622 (-1.565,0.300)
MPA	-0.870* (-1.570,-0.184)	-1.223* (-2.287,-0.180)	-0.623 (-1.556,0.293)
LPA	-0.871* (-1.581,-0.166)	-1.223* (-2.303,-0.171)	-0.623 (-1.550,0.278)
SED	-0.871* (-1.568,-0.170)	-1.224* (-2.302,-0.163)	-0.623 (-1.530,0.299)
**NIE**			
VPA	-0.137* (-0.296,-0.012)	-0.084 (-0.322,0.115)	-0.190* (-0.425,-0.020)
MVPA	-0.127* (-0.278,-0.012)	-0.061 (-0.272,0.116)	-0.192* (-0.422,-0.029)
MPA	-0.065 (-0.166,0.006)	-0.016 (-0.136,0.088)	-0.120 (-0.303,0.002)
LPA	-0.037 (-0.122,0.015)	-0.004 (-0.089,0.072)	-0.081 (-0.244,0.023)
SED	-0.004 (-0.060,0.049)	-0.046 (-0.199,0.053)	0.026 (-0.051,0.149)
**NDE**			
VPA	-0.733* (-1.429,-0.037)	-1.139* (-2.191,-0.087)	-0.431 (-1.362,0.501)
MVPA	-0.742* (-1.440,-0.045)	-1.162* (-2.218,-0.106)	-0.430 (-1.362,0.503)
MPA	-0.805* (-1.504,-0.105)	-1.208* (-2.271,-0.144)	-0.504 (-1.437,0.429)
LPA	-0.834* (-1.533,-0.134)	-1.219* (-2.284,-0.153)	-0.543 (-1.473,0.387)
SED	-0.867* (-1.566,-0.168)	-1.177* (-2.244,-0.111)	-0.649 (-1.579,0.282)
**CDE**			
VPA	-0.722* (-1.419,-0.025)	-1.133* (-2.188,-0.079)	-0.444 (-1.374,0.486)
MVPA	-0.740* (-1.438,-0.043)	-1.167* (-2.223,-0.112)	-0.426 (-1.359,0.505)
MPA	-0.788* (-1.489,-0.087)	-1.165* (-2.238,-0.092)	-0.520 (-1.451,0.412)
LPA	-0.834* (-1.533,-0.134)	-1.223* (-2.291,-0.155)	-0.543 (-1.472,0.386)
SED	-0.854* (-1.554,-0.153)	-1.160* (-2.224,-0.096)	-0.576 (-1.512,0.360)
**PM**			
VPA	0.157	-	-
MVPA	0.146	-	-
MPA	-	-	-
LPA	-	-	-
SED	-	-	-
**PE**			
VPA	0.170	-	-
MVPA	0.149	-	-
MPA	-	-	-
LPA	-	-	-
SED	-	-	-

*p < 0.050. Notes: numbers are estimates for association coefficients (95% confidence intervals). Models for all were adjusted for sex and age. Sex-specific models were adjusted for age. TE, Total Effect; NIE, Natural Indirect Effect; NDE, Natural Direct Effect; CDE, Controlled Direct Effect; PM, Proportion Mediated; PE, Proportion Eliminated; VPA, vigorous physical activity (per 15 min); MVPA, moderate-to-vigorous physical activity (per 15 min); MPA, moderate physical activity (per 15 min); LPA, light physical activity (per 60 min); SED, sedentary time (per 60 min)

Table S6 Fathers' education-related social gradient in psychosomatic symptoms and possible mediation via out-of-school physical activity intensities and sedentary time

Mediators	All	Male	Female
**TE**			
VPA	-0.922* (-1.525,-0.313)	-1.227* (-2.170,-0.302)	-0.705 (-1.515,0.115)
MVPA	-0.922* (-1.510,-0.298)	-1.227* (-2.161,-0.286)	-0.706 (-1.532,0.111)
MPA	-0.922* (-1.541,-0.320)	-1.228* (-2.190,-0.282)	-0.709 (-1.519,0.104)
LPA	-0.923* (-1.539,-0.316)	-1.227* (-2.181,-0.285)	-0.709 (-1.535,0.096)
SED	-0.923* (-1.528,-0.313)	-1.228* (-2.154,-0.300)	-0.708 (-1.498,0.102)
**NIE**			
VPA	-0.094* (-0.215,-0.005)	-0.037 (-0.173,0.060)	-0.173* (-0.393,-0.020)
MVPA	-0.082* (-0.194,-0.005)	-0.018 (-0.125,0.050)	-0.179* (-0.401,-0.030)
MPA	-0.037 (-0.110,0.007)	0.001 (-0.051,0.050)	-0.116* (-0.279,-0.003)
LPA	-0.024 (-0.089,0.018)	0.013 (-0.051,0.098)	-0.087 (-0.241,0.014)
SED	-0.002 (-0.043,0.032)	-0.017 (-0.140,0.071)	0.017 (-0.045,0.115)
**NDE**			
VPA	-0.829* (-1.436,-0.221)	-1.190* (-2.100,-0.280)	-0.532 (-1.346,0.282)
MVPA	-0.840* (-1.449,-0.230)	-1.209* (-2.126,-0.293)	-0.527 (-1.342,0.287)
MPA	-0.885* (-1.495,-0.275)	-1.229* (-2.151,-0.307	-0.593 (-1.407,0.222)
LPA	-0.898* (-1.509,-0.288)	-1.241* (-2.162,-0.319)	-0.622 (-1.436,0.192)
SED	-0.921* (-1.530,-0.312)	-1.211* (-2.136,-0.285)	-0.724 (-1.538,0.090)
**CDE**			
VPA	-0.824* (-1.433,-0.216)	-1.216* (-2.128,-0.303)	-0.546 (-1.359,0.267)
MVPA	-0.839* (-1.448,-0.229)	-1.197* (-2.113,-0.281)	-0.522 (-1.337,0.293)
MPA	-0.872* (-1.483,-0.260)	-1.252* (-2.183,-0.322)	-0.615 (-1.428,0.199)
LPA	-0.898* (-1.509,-0.288)	-1.267* (-2.191,-0.343)	-0.627 (-1.440,0.186)
SED	-0.906* (-1.516,-0.295)	-1.211* (-2.135,-0.287)	-0.662 (-1.481,0.157)
**PM**			
VPA	0.102	-	-
MVPA	0.089	-	-
MPA	-	-	-
LPA	-	-	-
SED	-	-	-
**PE**			
VPA	0.106	-	-
MVPA	0.091	-	-
MPA	-	-	-
LPA	-	-	-
SED	-	-	-

*p < 0.050. Notes: numbers are estimates for association coefficients (95% confidence intervals). Models for all were adjusted for sex and age. Sex-specific models were adjusted for age. TE, Total Effect; NIE, Natural Indirect Effect; NDE, Natural Direct Effect; CDE, Controlled Direct Effect; PM, Proportion Mediated; PE, Proportion Eliminated; VPA, vigorous physical activity (per 15 min); MVPA, moderate-to-vigorous physical activity (per 15 min); MPA, moderate physical activity (per 15 min); LPA, light physical activity (per 60 min); SED, sedentary time (per 60 min)

Table S7 Mothers' education-related social gradient in stress and possible mediation via out-of-school physical activity intensities and sedentary time

Mediators	All	Male	Female
**TE**			
VPA	-1.391* (-2.083,-0.688)	-1.827* (-2.881,-0.760)	-1.096* (-2.025,-0.167)
MVPA	-1.390* (-2.105,-0.686)	-1.827* (-2.903,-0.769)	-1.097* (-2.023,-0.173)
MPA	-1.390* (-2.100,-0.699)	-1.828* (-2.932,-0.759)	-1.098* (-2.014,-0.205)
LPA	-1.391* (-2.100,-0.698)	-1.828* (-2.921,-0.756)	-1.098* (-2.041,-0.166)
SED	-1.391* (-2.093,-0.677)	-1.828* (-2.944,-0.763)	-1.097* (-2.017,-0.164)
**NIE**			
VPA	-0.096 (-0.225,0.009)	0.054 (-0.098,0.239)	-0.264* (-0.496,-0.088)
MVPA	-0.058 (-0.169,0.034)	0.076 (-0.044,0.248)	-0.228* (-0.444,-0.066)
MPA	-0.003 (-0.070,0.061)	0.048 (-0.069,0.194)	-0.099 (-0.251,0.002)
LPA	-0.015 (-0.076,0.026)	-0.009 (-0.112,0.071)	-0.077 (-0.221,0.028)
SED	0.014 (-0.021,0.070)	-0.007 (-0.106,0.067)	0.021 (-0.059,0.127)
**NDE**			
VPA	-1.296* (-1.982,-0.609)	-1.881* (-2.917,-0.845)	-0.832 (-1.755,0.091)
MVPA	-1.333* (-2.020,-0.646)	-1.903* (-2.945,-0.860)	-0.869 (-1.790,0.052)
MPA	-1.387* (-2.076,-0.699)	-1.876* (-2.928,-0.824)	-0.999* (-1.918,-0.081)
LPA	-1.376* (-2.064,-0.687)	-1.818* (-2.862,-0.774	-1.021* (-1.937,-0.105)
SED	-1.405* (-2.095,-0.715)	-1.821* (-2.868,-0.775)	-1.118* (-2.038,-0.197)
**CDE**			
VPA	-1.260* (-1.950,-0.569)	-1.955* (-3.015,-0.896)	-0.919* (-1.838,-0.001)
MVPA	-1.291* (-1.982,-0.601)	-1.969* (-3.035,-0.903)	-0.950* (-1.868,-0.032)
MPA	-1.326* (-2.021,-0.632)	-1.894* (-2.973,-0.816)	-1.009* (-1.927,-0.091)
LPA	-1.383* (-2.073,-0.694)	-1.958* (-3.012,-0.905)	-1.033* (-1.948,-0.118)
SED	-1.409* (-2.102,-0.716)	-1.820* (-2.867,-0.774)	-1.155* (-2.085,-0.224)
**PM**			
VPA	-	-	-
MVPA	-	-	-
MPA	-	-	-
LPA	-	-	-
SED	-	-	-
**PE**			
VPA	-	-	-
MVPA	-	-	-
MPA	-	-	-
LPA	-	-	-
SED	-	-	-

*p < 0.050. Notes: numbers are estimates for association coefficients (95% confidence intervals). Models for all were adjusted for sex and age. Sex-specific models were adjusted for age. TE, Total Effect; NIE, Natural Indirect Effect; NDE, Natural Direct Effect; CDE, Controlled Direct Effect; PM, Proportion Mediated; PE, Proportion Eliminated; VPA, vigorous physical activity (per 15 min); MVPA, moderate-to-vigorous physical activity (per 15 min); MPA, moderate physical activity (per 15 min); LPA, light physical activity (per 60 min); SED, sedentary time (per 60 min)

Table S8 Mothers' education-related social gradient in psychosomatic symptoms and possible mediation via out-of-school physical activity intensities and sedentary time

Mediators	All	Male	Female
**TE**			
VPA	-1.108* (-1.743,-0.480)	-1.477* (-2.449,-0.534)	-0.847* (-1.656,-0.023)
MVPA	-1.108* (-1.730,-0.478)	-1.478* (-2.460,-0.494)	-0.847* (-1.649,-0.034)
MPA	-1.107* (-1.744,-0.495)	-1.479* (-2.474,-0.510)	-0.848* (-1.663,-0.025)
LPA	-1.108* (-1.755,-0.486)	-1.479* (-2.477,-0.525)	-0.849* (-1.676,-0.032)
SED	-1.108* (-1.733,-0.480)	-1.478* (-2.460,-0.518)	-0.847* (-1.665,-0.016)
**NIE**			
VPA	-0.073 (-0.179,0.007)	0.042 (-0.069,0.196)	-0.203* (-0.391,-0.058)
MVPA	-0.043 (-0.129,0.027)	0.050 (-0.034,0.185)	-0.182* (-0.360,-0.049)
MPA	-0.001 (-0.051,0.046)	0.010 (-0.097,0.120)	-0.084 (-0.210,0.003)
LPA	-0.013 (-0.063,0.023)	-0.013 (-0.107,0.056)	-0.072 (-0.203,0.024)
SED	0.004 (-0.028,0.039)	0.002 (-0.060,0.054)	0.017 (-0.049,0.108)
**NDE**			
VPA	-1.035* (-1.636,-0.434)	-1.519* (-2.428,-0.610)	-0.643 (-1.449,0.162)
MVPA	-1.065* (-1.668,-0.463)	-1.528* (-2.447,-0.609)	-0.665 (-1.468,0.139)
MPA	-1.106* (-1.709,-0.502)	-1.489* (-2.418,-0.559)	-0.764 (-1.564,0.036)
LPA	-1.095* (-1.700,-0.491)	-1.466* (-2.388,-0.544)	-0.777 (-1.575,0.022)
SED	-1.111* (-1.716,-0.507)	-1.480* (-2.404,-0.556)	-0.864* (-1.667,-0.062)
**CDE**			
VPA	-1.026* (-1.631,-0.421)	-1.707* (-2.637,-0.777)	-0.712 (-1.515,0.090)
MVPA	-1.041* (-1.647,-0.435)	-1.683* (-2.624,-0.743)	-0.739 (-1.539,0.062)
MPA	-1.041* (-1.650,-0.432)	-1.521* (-2.474,-0.568)	-0.774 (-1.574,0.025)
LPA	-1.093* (-1.699,-0.488)	-1.591* (-2.522,-0.661)	-0.804* (-1.601,-0.006)
SED	-1.136* (-1.743,-0.528)	-1.481* (-2.405,-0.557)	-0.892* (-1.703,-0.081)
**PM**			
VPA	-	-	-
MVPA	-	-	-
MPA	-	-	-
LPA	-	-	-
SED	-	-	-
**PE**			
VPA	-	-	-
MVPA	-	-	-
MPA	-	-	-
LPA	-	-	-
SED	-	-	-

*p < 0.050. Notes: numbers are estimates for association coefficients (95% confidence intervals). Models for all were adjusted for sex and age. Sex-specific models were adjusted for age. TE, Total Effect; NIE, Natural Indirect Effect; NDE, Natural Direct Effect; CDE, Controlled Direct Effect; PM, Proportion Mediated; PE, Proportion Eliminated; VPA, vigorous physical activity (per 15 min); MVPA, moderate-to-vigorous physical activity (per 15 min); MPA, moderate physical activity (per 15 min); LPA, light physical activity (per 60 min); SED, sedentary time (per 60 min)
